# On-Site Medical Management of Avalanche Victims—A Narrative Review

**DOI:** 10.3390/ijerph181910234

**Published:** 2021-09-29

**Authors:** Simon Rauch, Giacomo Strapazzon, Hermann Brugger

**Affiliations:** 1 Institute of Mountain Emergency Medicine, Eurac Research, Via Ipazia 2, 39100 Bolzano, Italy; giacomo.strapazzon@eurac.edu (G.S.); hermann.brugger@eurac.edu (H.B.); 2 Department of Anesthesiology and Intensive Care Medicine, “F. Tappeiner” Hospital, 39012 Merano, Italy

**Keywords:** avalanche, avalanche burial, cardiac arrest, emergency medicine, hypothermia, mountain rescue

## Abstract

Avalanche accidents are common in mountain regions and approximately 100 fatalities are counted in Europe each year. The average mortality rate is about 25% and survival chances are mainly determined by the degree and duration of avalanche burial, the patency of the airway, the presence of an air pocket, snow characteristics, and the severity of traumatic injuries. The most common cause of death in completely buried avalanche victims is asphyxia followed by trauma. Hypothermia accounts for a minority of deaths; however, hypothermic cardiac arrest has a favorable prognosis and prolonged resuscitation and extracorporeal rewarming are indicated. In this article, we give an overview on the pathophysiology and on-site management of avalanche victims.

## 1. Introduction and Epidemiology

Snow avalanches are common events in mountainous regions worldwide. About 100 avalanche fatalities are counted per year in Europe [1], while avalanches inflict even higher death tolls in developing countries (e.g., 284 fatalities in South East Anatolia, Turkey in 1992; 135 in Kashmir, Pakistan in 2012) [2], but comprehensive data are missing. Most avalanche accidents occur during recreational activities in the mountains in uncontrolled terrain, while occupational-related avalanche accidents are much less frequent [1,3]. The number of avalanche accidents involving winter recreationists has increased in recent decades due to the increasing popularity of winter outdoor activities [4]. However, in contrast to this development, the number of fatalities has remained relatively stable since the 1980s [1]. In Tyrol, Austria, two-thirds of avalanche accidents occurred on days with hazard grades 2 (triggering of an avalanche is possible with high additional loads) and 3 (triggering is possible even with low additional loads) on slope angles of 35–40 [5]. Most avalanches were triggered in open areas without trees above 2500 m, although >15% occurred below timberline. The highest incidence of avalanche accidents was between 10 a.m. and 3 p.m. [5].

The ongoing climate change may influence the frequency and change the types of snow avalanches, affecting the grade of avalanche burial, pattern of injuries, and survival rates [6]. A wetter and warmer snow climate might lead to higher snow densities and this will likely interfere with the respiration of completely buried victims and increase the rate of asphyxia [7]. Moreover, blunt trauma and secondary injuries will likely become more frequent as terrain roughness is expected to rise and snow cover to become thinner [6].

In this narrative review we give an overview of the pathophysiological aspects and current state-of-the art of on-site treatment of avalanche victims.

## 2. Pathophysiology

Avalanche accidents have an average mortality rate of 25% [8]. Survival chances are largely determined by the degree and duration of avalanche burial, the patency of the airway, the presence of an air pocket, snow characteristics, and the severity of traumatic injuries.

The chance of survival after complete burial (head and chest below the snow) for an uninjured victim is about 50%. If only partially buried (head and chest out of the snow), chance of survival is >95% [8,9,10].

**Figure 1:**
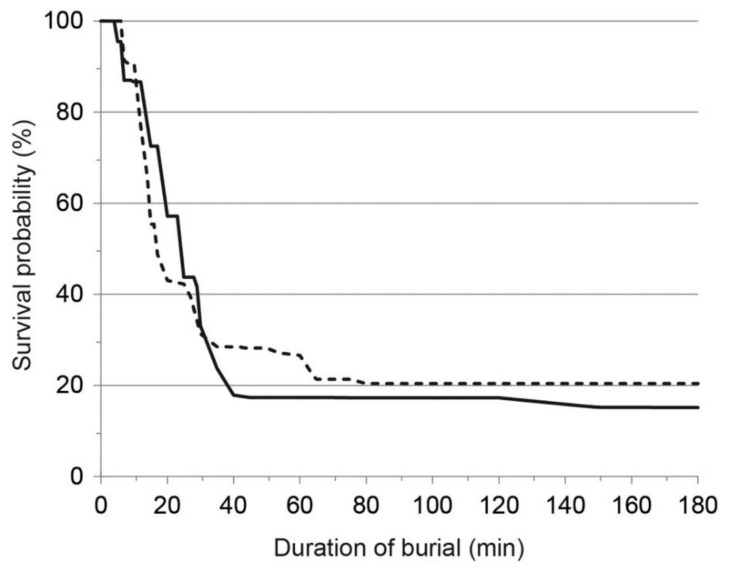
Survival curve for Austria (solid line) and Switzerland (dashed line) for completely buried victims between 2005 and 2013. Reprinted with permission from [12].

In case of complete burial, the chance of survival is highly time-dependent (Figure 1) [11,12]. The survival rate is around 90% if the victim is rescued within the first 15–20 min (‘survival phase’). Thereafter, survival probability drops sharply to about 30% (~35 min after burial). The main cause of death in this second phase is asphyxia (‘asphyxia phase’); asphyxia causes about 75% of overall avalanche deaths. Asphyxiation during avalanche burial mainly occurs due to airway obstruction with snow, ice mask formation, impaired chest expansion and ventilation because of the weight and compaction of snow, and oxygen deprivation due to rebreathing of expired air. The onset of asphyxia depends also on snow characteristics [7]. From 35 to 90 min, the avalanche survival curve flattens (‘latent phase’). In this phase, survival is possible only if the airway is patent, and it is favored by the presence of an air pocket or mid-low snow density [12,13]. The combination of severe hypothermia, hypoxia, and hypercapnia (triple H syndrome) [14] is the usual cause of death in uninjured victims during the latent and the long-term survival phase (>90 min) [14,15,16]. Rebreathing expired air during avalanche burial results in a progressive decrease in the fraction of inspired oxygen and an increase in the fraction of inspired carbon dioxide, leading to hypoxia and hypercapnia, respectively [17,18]. Accidental hypothermia is the main cause of death in only about 1% of completely buried avalanche victims, but it should be suspected in survivors who do not die of asphyxia within 35 min, especially in victims buried for >60 min [19,20,21]. With cooling, there is a decrease in oxygen consumption of ~6% for every 1 °C reduction in core temperature [22]. A mean core cooling rate of 3 °C/h has been calculated for the entire time between avalanche burial and hospital admission [23], yet, the individual cooling rates during snow burial vary widely, from 0.1 °C/h to 9 °C/h [24,25]. It generally takes at least 60 min after avalanche burial to reach a core temperature <30 °C [20].

Trauma accounts for less than 25% of avalanche deaths in North America and Europe [10]. Traumatic deaths are associated with a wide range of injuries that depend on topographic differences in terrain and snow characteristics [6]. Avalanche victims can sustain virtually any type of injury during the often turbulent descent in an avalanche; severe injuries are frequently caused by collisions with trees or rocks [26].

## 3. On-Site Management

### 3.1. Guidelines for On-Site Management

The first algorithm for on-site triage of avalanche victims with asystole was published in 1996 [27]. Recommendations for on-site care of avalanche victims were adopted by the International Commission for Mountain Emergency Medicine (ICAR MedCom) in 2002 [28] and updated in 2013 [2]. In 2010, guidelines were approved by the International Liaison Committee for Resuscitation (ILCOR) and incorporated into the European Resuscitation Council (ERC) and American Heart Association (AHA) guidelines [29,30]. In 2015, the ERC dedicated a section of the resuscitation guidelines in special circumstances to mountain emergency medicine and avalanche rescue [31]. The Wilderness Medical Society (WMS) published Practice Guidelines for Prevention and Management of Avalanche and Nonavalanche Snow Burial Accidents in 2017 [10]. Finally, in 2021, the ERC updated their guidelines [20].

### 3.2. Companion and Organized Rescue

The highly time dependent survival from complete avalanche burial highlights the importance of immediate rescue by uninjured companions [32]. The likelihood of survival can be four times higher if the victim is rescued by uninjured companions compared to organized rescue [32]. Once at the scene, it takes at least 3–5 min to locate a completely buried person. Once located, in a simulation study with manikins buried at a depth of 1 m, it took an average of 7 min to free the airway, plus a further 3 min to initiate cardiopulmonary resuscitation (CPR). The number of rescuers (one compared to two) and the body position of the buried manikin had no significant influence on extrication times [33]. Since asphyxia is the most frequent cause of death in the early phase of burial, basic life support by first responders must include ventilations and chest compressions (compression-only CPR is not recommended [2].

Organized rescue differs from companion rescue because the response time is typically longer, resources are more available, and group size is larger. For avalanche rescue, if conditions allow, helicopters should be given priority over ground rescue teams, as they are faster, safer, and more efficient. Helicopters can also be used to search from the air with avalanche transceivers and RECCO^®^ (see below) [34]. However, air rescue operations may be restricted by adverse weather conditions with low visibility and wind.

An organized search for a completely buried avalanche victim can rely on visual and acoustic methods, avalanche transceivers, avalanche dogs, the RECCO^®^ rescue system, and probing. Companions and rescuers should immediately search for clues on the surface and acoustic signs (calling out and listening for victims’ cries for help). Rescue dogs are trained to detect buried victims by scent. The initial dog search should be with the handler before the terrain is contaminated by helicopter fumes. The search continues with avalanche transceivers and the RECCO^®^ system, if available [34]. The RECCO^®^ technology system is a two-part system, featuring an active detector, carried by the rescuer, and a passive reflector diode, embedded into the victim’s clothing. It is used by more than 600 rescue organizations worldwide, especially in ski areas [34]. If attempts with avalanche transceivers and RECCO^®^ fail, rescuers should begin a probe-line search. The initial probing depth should be limited to 1.5 m and the ‘Three Holes per Step’ or the ‘Slalom’ probing techniques should be used [10]. A probe line leader is necessary to ensure accuracy and effectiveness. Professional healthcare personnel should be at the scene to treat victims during and after extrication.

Every ground or air rescuer who enters avalanche terrain must be properly equipped with an avalanche transceiver and, ideally, an avalanche airbag. Medical equipment should be protected from the cold. Electronic instruments should have full batteries. Rescue equipment should include rescue bags, which are similar to sleeping bags but modified for rescue use, or other insulating layers, aluminum blankets, chemical heat packs, a thermometer suitable for measuring core temperature, and a cardiac monitor/defibrillator [2].

### 3.3. Extrication, Initial Assessment and Monitoring

In an organized avalanche search and rescue (SAR) operation, the duration of victim burial usually exceeds 35 min. Victims can be expected to be mildly or moderately hypothermic. For this reason, extrication and the initial assessment should be carried out as carefully, rather than as rapidly, as possible. Rescuers should first assess the position of a victim, then dig a channel towards their head. The rescuer who uncovers the face should check whether there is an air pocket in front of the mouth and nose, noting whether the airway is open or is blocked by snow or debris. Ideally, these observations should be made by a rescuer trained in advanced life support (ALS) or an emergency physician. Knowing whether the airway was patent or obstructed and if there was an air pocket is crucial for decisions concerning resuscitation and transport. If the position of the victim allows, the first assessment of the airway and vital signs should be made before complete extrication. An ECG should be obtained as soon as possible, before removal and transport of the victim, as it allows for the detection of arrhythmias provoked by movement of the patient [35]. Core temperature should be measured using an esophageal probe or a low reading thermistor-based epitympanic thermometer [36]. Esophageal temperature measurement correlates well with cardiac temperature. An esophageal probe placed with the distal end in the lower third of the esophagus is considered the gold standard for patients in cardiac arrest or in whom advanced airway management is necessary [37]. Epitympanic measurement using a thermistor is a reliable alternative in patients not in cardiac arrest but may register a much lower temperature than actual core temperature if the environment is very cold [38]. The probe must be well insulated and the external auditory canal must be free of snow or water. Epitympanic probes not manufactured for outdoor use should not be used at the scene [36]. Pulse oximetry is not mandatory, as it may be inaccurate with cold exposure due to peripheral vasoconstriction.

As soon as a victim is extricated, a first assessment should be made to look for vital signs and evident injuries. Cardiac activity and core temperature should be continuously monitored throughout the rescue for early detection of after-drop or circum-rescue collapse. If a defibrillator is available, defibrillator pads should be put in position. The victim should be protected from cold and wind [2,20].

### 3.4. Trauma Management

Chest and head trauma are the most frequent injuries in avalanches, while spinal, abdominal, and limb injuries are less frequent [26]. General concepts of trauma management also apply to avalanche victims: Current resuscitation guidelines emphasize early hemorrhage control, damage-control resuscitation, advanced airway management if indicated, stabilization of injuries, and prompt evacuation to definitive care [39,40]. Spinal motion restriction, splinting limb fractures, and administration of effective analgesia should be performed during on-site management and transport. In severe head trauma, early intubation and normocapnic ventilation is recommended. Tourniquets can be life-saving in exsanguinating limb injuries. Immediate chest decompression is mandatory for tension pneumothorax. For pneumo- or hemothorax, a thoracostomy tube should be considered, particularly before evacuation by helicopter if a climb in altitude is expected and the victim is intubated.

In victims of traumatic cardiac arrest, survival is low; prolonged CPR is associated with poor neurological outcomes.

### 3.5. Airway Management and Ventilation

For unconscious avalanche victims, advanced airway management provides effective oxygenation, reducing the likelihood of aspiration. Endotracheal intubation can, rarely, provoke ventricular fibrillation in victims with moderate or severe hypothermia, usually at a core temperature < 30 °C [41,42]. As the evidence for this is mainly animal-based; the small risk is far outweighed by the advantages of airway control [43,44].

Whether ventilation in unconscious avalanche victims should target normocapnia (endtidal CO_2_ 35–45 mmHg) is controversial. Hypocapnia, (endtidal CO_2_ <35 mmHg) due to excessive ventilation or decreased metabolic production of CO_2,_ decreases cerebral blood flow due to vasoconstriction, which can induce arrhythmias as frequently as hypercapnia, especially in victims with hypothermia. Normoxia may protect against malignant arrhythmias, as it improves myocardial stability in asphyxiated as well as in severely hypothermic victims. It seems likely that adequate oxygenation might help to reduce the risk of circum-rescue collapse.

Endotracheal intubation requires training and practice; therefore, it should be done only by qualified rescuers with a high tracheal intubation success rate [45]. Placement of supraglottic devices is easier and safer than endotracheal intubation [46]. For rescuers who are not experienced in advanced airway management, ventilation is most effective with mouth-to-mask or bag–valve–mask techniques. For a survivor with an unsecured airway, hospital transport should be expedited for advanced airway management [22].

### 3.6. Management of Moderate and Severe Hypothermia

Moderate or severe hypothermia should be suspected in a cold and unconscious avalanche victim. Extrication should be carried out gently, without unnecessary movement and immobilization, onto a horizontally positioned stretcher in order to avoid after-drop and circum-rescue collapse due to ventricular fibrillation. If the victim is neither shivering nor moving, exposure to cold and wind after extrication can cause a rapid increase in the cooling rate, with an increased risk of ventricular arrhythmias and cardiac arrest, particularly if consciousness is impaired [47]. Pre-hospital insulation and application of external heat immediately after extrication is mandatory for all immobile avalanche victims. Multi-layer packaging of the victim should include an external heat source, such as chemical heat packs, applied to the chest but not directly to the skin because of the risk of burns. The victim should then be wrapped in the thickest available dry insulation, usually an insulated rescue bag or sleeping bag, with a vapor barrier outer layer, such as an aluminum blanket or bubble wrap [48]. Removing wet clothes increases victim comfort but results in rapid cooling in a cold or windy environment and is not necessary if the victim can be properly insulated and a vapor barrier placed [10].

If an avalanche victim is unconscious when extricated, their core temperature should be measured early to distinguish between severe hypothermia and other causes of unconsciousness, such as asphyxia or traumatic brain injury.

Severely hypothermic avalanche victims who present with a core temperature <30 °C, systolic blood pressure <90 mmHg, ventricular arrhythmias, or any other cardiac instability should ideally be transported directly to a center offering extracorporeal life support (ECLS). This action is not necessarily for extracorporeal rewarming but to have the optimal treatment at hand in case ECLS treatment becomes necessary [20,49].

### 3.7. Management of Patients in Cardiac Arrest

#### 3.7.1. Management Algorithm

**Figure 2:**
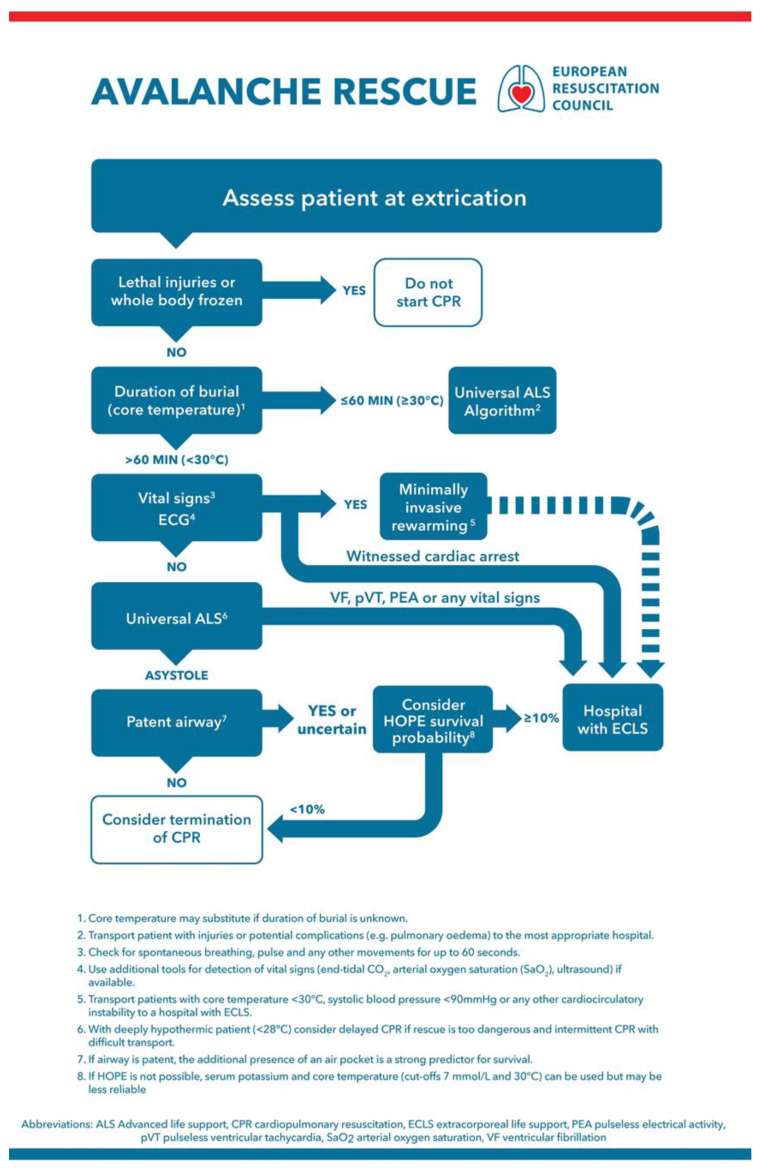
Avalanche accident algorithm. Management of completely buried victims. Reprinted with permission from [20].

In the section on cardiac arrest in special circumstances of the current ERC guidelines 2021, the management of avalanche victims is covered and a management algorithm included (Figure 2) [20].

Detecting signs of life may be difficult in severely hypothermic avalanche victims, as respiration and pulse may be very slow, irregular, and faint. Vital signs should therefore be checked for up to 1 min rather than the 10 s recommended for normothermic victims [20]. Unless an indication for withholding resuscitation exists (see below), CPR should be commenced by starting with five ventilations, as hypoxia is the most likely cause of cardiac arrest [20]. If duration of burial is <60 min and core temperature is <30 °C, cardiac arrest is likely due to trauma or asphyxia; standard ALS, as for normothermic patients, should therefore be performed. If duration of burial is >60 min, the core temperature is <30 °C, and the victim has a patent airway at extrication, cardiac arrest may be attributed to hypothermia. In this case, CPR should be continued, and the patient transported to a hospital with ECLS capability [20]. The destination hospital should be contacted in advance to ensure the availability of ECLS. An avalanche victim who has a perfusing rhythm at extrication and subsequently had a witnessed cardiac arrest has a good chance of neurologically intact survival [50]. The rate of chest compressions and ventilation should be the same as in standard BLS, with minimal interruptions.

#### 3.7.2. Intermittent CPR

During difficult and long transport of patients with ongoing CPR, mechanical chest compression devices should be used, if available [10,51]. If continuous mechanical or manual CPR is not possible in a patient with a core temperature <28 °C or with unknown core temperature but unequivocal hypothermic cardiac arrest, intermittent CPR can be performed with ≥5 min of CPR alternated with ≤5-min interruptions. If core temperature is <20 °C, interruptions can be ≤10 min [10,20].

#### 3.7.3. Drugs and Defibrillation

For volume resuscitation and administration of drugs, intravenous, or intraosseous access should be obtained [45]. Obtaining peripheral intravenous access can be difficult if the victim is hypothermic with peripheral vasoconstriction and centralized circulation. It is usually easier to obtain intraosseous access under these circumstances.

In a hypothermic victim, aggressive volume replacement is usually not indicated, as hypothermia causes cardiovascular depression. If fluids are administered, e.g., for volume replacement in the hypotensive trauma patient, infused fluids should be warmed to ~42 °C. Since this can be difficult in the field, fluid infusion should, if possible, be delayed until the victim is loaded into a heated ambulance or helicopter.

The hypothermic heart may be unresponsive to cardioactive drugs, attempted electrical pacing and defibrillation. In a hypothermic victim, particularly if core temperature is <30 °C, administration of ALS drugs is therefore controversial. For a victim in cardiac arrest, vasopressors such as adrenaline are intended to augment myocardial blood flow and increase the return of spontaneous circulation. Adrenaline (1 mg) improved the rate of survival to hospital admission and long-term survival (to 3 months) but did not improve favorable neurological outcome in normothermic cardiac arrest [52,53]. Yet, the effectiveness of vasopressors has never been demonstrated in a hypothermic victim with core temperature <30 °C. The current ERC guidelines recommend that adrenaline should be withheld if the core temperature is <30 °C; administration intervals for adrenaline should be increased to 6–10 min if the core temperature is 30–35 °C. As normothermia (>35 °C) is approached, standard drug protocols become effective again [20].

The benefit of antiarrhythmic drugs in hypothermic victims is also unclear. Many arrhythmias, including atrioventricular blocks, atrial fibrillation, and nodal rhythms are benign, reversible with rewarming, requiring no further treatment if perfusion is adequate. Bradycardia may be physiologic in severe hypothermia. Cardiac pacing generally is not required unless the bradycardia persists despite rewarming to 32 to 35 °C.

Most intravenous drugs for induction of anesthesia can cause cardiovascular depression. Ketamine is likely to be safe in a hypothermic victim [54], but the sympathomimetic effects could theoretically cause problems for an irritable heart [55]. Neuromuscular transmission decreases in hypothermia, and sensitivity to non-depolarizing muscle relaxants increases [56,57]. If depolarizing muscle relaxants, such as suxamethonium, are used for paralysis to obtain favorable intubating conditions, the potential to increase serum potassium should be taken into account [58]. This may affect subsequent resuscitation or advanced rewarming decisions. Hypothermia also reduces the systemic clearance of cytochrome P450, which is involved in the metabolism of many drugs, such as propofol and ketamine. Use of small doses is therefore desirable for induction of anesthesia [22].

Providers should institute continuous ECG monitoring, place defibrillator pads during rescue and transport, and be prepared to start CPR. The hypothermic heart may not only be unresponsive to cardioactive drugs but also to attempted electrical pacing and defibrillation. The ERC guidelines recommend up to three defibrillation attempts with a core temperature <30 °C and, if VF persists after three shocks, delaying further attempts until core temperature is >30 °C [20].

#### 3.7.4. Prognostication of Successful Rewarming

On hospital admission, the HOPE (Hypothermia Outcome Prediction after ECLS) score should be used for in-hospital prognostication of successful rewarming [20,59,60]. HOPE provides a prediction of the survival probability in hypothermic cardiac arrest patients undergoing ECLS rewarming and includes the following variables: age, sex, presence or absence of asphyxia, CPR duration, serum potassium, and core body temperature. A cutoff of 10% has been proposed to decide which hypothermic patients in cardiac arrest would benefit or not from ECLS rewarming. The negative predictive value of a HOPE probability <10% was 97%, with the area under the receiver operating characteristic curve of 0.825 [60]. Of note, when calculating the HOPE score (https://www.hypothermiascore.org, accessed on 18 September 2021), “hypothermia with asphyxia” must be selected for all buried victims who were already in cardiac arrest at the time of extrication, while “hypothermia without asphyxia” must be entered for partially or non-buried victims and cases of witnessed cardiac arrest. The traditional triage with serum potassium and core temperature (cut-offs 7 mmol/L and 30 °C, respectively) can be used if the HOPE score cannot be calculated but may be less reliable [61].

#### 3.7.5. Training in Avalanche Rescue and the Avalanche Victim Resuscitation Checklist

Although existing guidelines [2,20] for avalanche victims in cardiac arrest are simple, making the right decisions can be very challenging in the stressful environment of an avalanche. A study in the European Alps showed poor adherence with the ICAR MEDCOM guidelines for avalanche victims with out-of-hospital cardiac arrest in the period 1987–2009 [62]. Data of key parameters, such as the extent and duration of burial, core temperature at the scene, and patency of the airway, were incomplete. Overall survival rate was very low, and initiation of CPR was lower than expected for patients with long burials and patent airways, with the reasons to initiate or withhold resuscitation remaining unclear in the majority of cases [62]. Deficiencies in awareness of the guidelines by bystanders, first responders, and hospital personnel, and the transfer of essential information from the accident site to hospital may have been partially responsible for poor outcomes. About 75% of BLS and ALS providers, and members of mountain rescue services working in areas in which they were likely to manage avalanche victims, at the scene or in hospital, had never participated in avalanche-specific rescue training [63].

The Avalanche Victim Resuscitation Checklist, introduced in 2015, should help rescuers to adhere to recommended medical management of avalanche victims by facilitating reliable data transfer from avalanche sites to hospitals [64]. The checklist is designed to be completed at the accident site and remains with the victim until hospital admission. Avalanche SAR teams and healthcare providers should periodically receive training in specific skills, such as pre-hospital core temperature measurement, insulation, and rewarming techniques. Protocols for the transport of avalanche victims to the most suitable hospital should be available to dispatchers. Ideally, a coordinator, who is an accidental hypothermia specialist, should be on call to assist with management of critical hypothermia victims [65].

#### 3.7.6. Termination of CPR

CPR should be considered futile in cardiac arrest with a burial time >60 min and additional evidence of an obstructed airway [10,20], or when an ECG asystole is detected. Moreover, CPR may be withheld or terminated in a victim without vital signs when the risk is unacceptable to the rescuer, the rescuer is exhausted, or when CPR cannot be performed due to extreme environmental conditions. CPR should be withheld if the victim has sustained injuries incompatible with life, such as decapitation, truncal transection, if the body is frozen solid, or a do-not-resuscitate order is evident [66].

## 4. Conclusions

Mortality rate of completely buried avalanche victims is about 50%. The most common cause of death is asphyxia followed by trauma, while hypothermic cardiac arrest is rare. Severe hypothermia has, however, a neuroprotective effect and hypothermic cardiac arrest is associated with a favorable outcome. An avalanche victim who had a witnessed cardiac arrest after extrication has a particularly good chance of neurologically intact survival. Avalanche resuscitation guidelines and the avalanche resuscitation checklist are helpful in differentiating non-hypothermic from hypothermic cardiac arrest, thereby optimizing patient and resource management. Periodic training of avalanche SAR teams is paramount for the optimal management of avalanche victims.
